# Mutual effects of ablation and ice temperature in a firn-free glacier: Observations from Aug 1st glacier on northeast Tibetan plateau

**DOI:** 10.1016/j.isci.2025.114456

**Published:** 2025-12-16

**Authors:** Guohua Liu, Rensheng Chen, Chuntan Han, Junfeng Liu, Zhangwen Liu, Yong Yang, Shuihai Guo, Xiqiang Wang, Yiwen Liu, Chanchan Gao

**Affiliations:** 1 College of Geography and Tourism, Hengyang Normal University, Hengyang, Hunan, China; 2 Key Laboratory of Ecological Safety and Sustainable Development in Arid Lands, Northwest Institute of Eco-Environment and Resources, Chinese Academy of Sciences, Lanzhou, Gansu, China; 3 Qilian Alpine Ecology and Hydrology Research Station, Key Laboratory of Ecological Safety and Sustainable Development in Arid Lands, Northwest Institute of Eco-Environment and Resources, Chinese Academy of Sciences, Lanzhou, Gansu, China

**Keywords:** Earth sciences, Earth-surface processes, Glacial processes

## Abstract

Glacier stability depends on the interaction between ice temperature and ablation, processes mediated by firn cover. Although firn-covered glaciers have been extensively investigated, firn-free glacier dynamics remain less clear. This study analyzes the coupled response of ice temperature and ablation to air temperature on the firn-free Aug 1st glacier in the northeastern Tibetan plateau. Results indicate that surface ice temperature is positively correlated with air temperature; however, without firn to store meltwater, latent heat uptake and runoff-related heat loss reduce the rate of surface warming. Increased ablation also limits heat penetration, encouraging active-layer thinning under stable internal ice temperatures. By contrast, ice temperature has little effect on ablation, which is governed mainly by air temperature. These results advance knowledge of glacier thermal behavior and underscore the importance of glacier type in modeling ice temperature evolution.

## Highlights

• Study focuses on a firn-free glacier and its thermal-ablation dynamics
• Absence of firn layer alters glacier ablation-ice temperature interactions
• Meltwater processes buffer englacial temperature rise
• Air temperature controls ablation; short-term ice temperature fluctuations do not

## Graphical abstract

**Figure:**
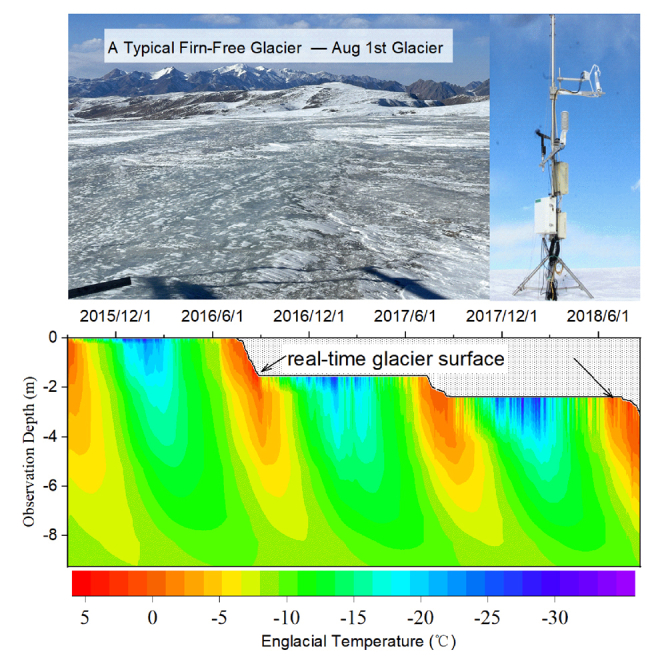

## Introduction

As glacier surface melting accelerates, heat transfer into the glacier interior has generally led to rising ice temperatures worldwide, weakening the inherent stability of glaciers.^1^^,^^2^^,^^3^^,^^4^^,^^5^ Nevertheless, modeling studies suggest that this warming trend is not universal—particularly for small glaciers, where ice temperatures may instead experience cooling under global warming.^6^^,^^7^

The firn layer plays a key role in regulating ice temperature because it prevents rapid meltwater runoff and provides storage conditions for meltwater infiltration and refreezing.^8^^,^^9^ With global warming, if the firn layer disappears completely, the glacier is no longer able to store and infiltrate meltwater, and the pathway for rapid diffusion of external heat to the interior of the glacier is lost.^6^ In addition, the energy consumed by glacier ablation and the energy lost by glacier meltwater runoff can lead to a decrease in englacial temperature.^10^ The earlier mentioned process is confirmed by the results of englacial temperature simulations of small glaciers, <0.5 km^2^, in the Alps, which show that the disappearance of the firn layer led to a decrease of approximately 1.0°C in 10 m englacial temperature during 1960∼2010.^6^ A combination of *in situ* measurements and a coupled ice-flow model simulated of the Laohugou No. 12 glacier also showed cooling trend of englacial temperature from 1971–2011.^7^

In the context of ongoing global climate warming, glacier responses to climate change will become more intense and complex,^11^ with even small processes potentially triggering a series of chain reactions leading to increasingly uncertain outcomes.^10^ A key feature of future glaciers will be the complete disappearance of accumulation areas; by the end of this century, accumulation areas may constitute less than 5% of the total glacier area under high-emission scenarios in the high Asian mountains.^12^ This means that firn layers of glaciers will disappear in the future, particularly in small glaciers in Asian high mountains. Once glaciers lose their accumulation areas, they undergo irreversible accelerated melting to extinction, even if warming stops.^13^ Under such circumstances, elucidating the role of firn layer disappearance in modulating englacial temperature, and in turn influencing ablation processes, becomes a critical scientific issue. Addressing this question is essential for advancing a more nuanced understanding of how glaciers without firn layers respond to ongoing climate change.

The Aug 1st glacier in the Qilian mountains experienced a heat wave in 2016. Taking this as an opportunity, this study aims to reveal the response of glacier ablation and ice temperature to air temperature based on observations of the Aug 1st glacier. By comparing the changes in glacier surface temperature and englacial temperature and the glacier ablation in 2016 and 2017, we analyzed the mutual feedback mechanism between the glacier ablation and ice temperature. The results of this study provide improved insights into glacier changes and help enhance the accuracy of glacier modeling and forecasting.

### Study site

**Figure 1:**
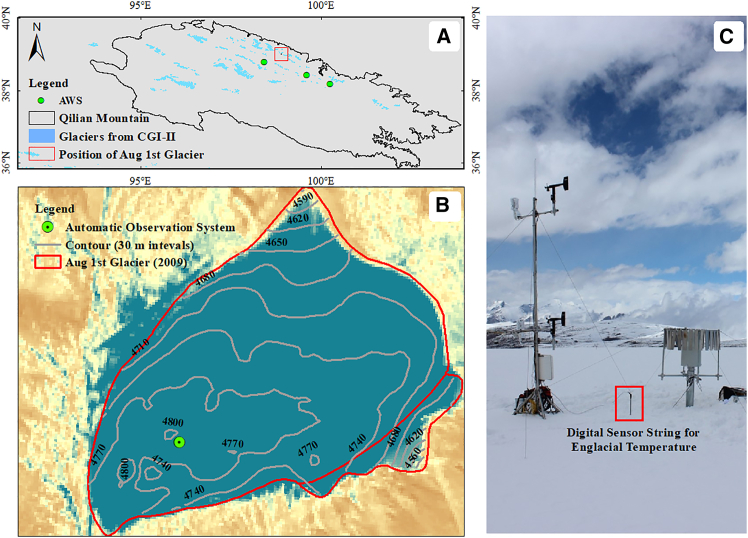
Aug 1st glacier in the Qilian mountains in northwestern China in 2020 (A) shows regional climate change measured by the China Meteorological Administration and the locations of three AWSs (green points), (B) shows the location of an automatic observation system (green circle), and (C) illustrates this observation system with an englacial temperature observation chain (red box) used to monitor surface and englacial temperature.

The Aug 1st glacier is an ice-cap glacier located in the Qilian mountains on the northeastern edge of the Qinghai-Tibet plateau (Figure 1A). Given its slow movement rate and flat topography, the Aug 1st glacier has long been a focal point for glacier observation, including in the Cryospheric Hydrometeorology Observation in the Hulu Catchment.^14^ According to the first Chinese Glacier Inventory,^15^ the glacier area was 2.81 km^2^ in 1956, and then it decreased to 2.3 km^2^ in 2021; however, the reduction was relatively moderate compared with those of other glaciers in the region. The glacier has an elevation range of 4,520∼4,828 m, and the maximum thickness of the Aug 1st glacier at its summit is 82 m. Previous modeling studies have shown that the equilibrium line of the Aug 1st glacier began to rise in 1980, reaching approximately 4,800 m by the early 21th century.^16^ During this period, the glacial accumulation area gradually decreased from 2.3 km^2^ to 0.5 km^2^. After 2010, for both the Aug 1st glacier and other glaciers in the region, the elevation of the equilibrium line almost exceeded that of the glaciers at the highest elevation, and the accumulation area of the glacier essentially disappeared. In particular, the Aug 1st glacier has completely lost its firn layer in recent years. Following the rapid melting of the thin layer of accumulated snow on the surface of the glacier during the melting season, the ice surface is completely exposed.

Currently, basic meteorological and glacier change monitoring equipment has been installed on the Aug 1st glacier. However, the observation period is relatively short. Therefore, when research on long-term climate change conducted, data from three nearby national meteorological stations from the China Meteorological Administration are needed. The locations of the meteorological stations on or near the glacier are shown in Figures 1A and 1B, with details shown in Table 1.

**Table 1:** Details of the weather stations on Aug 1st glacier and its surrounding areas

Id (as in Figure 1)	Lat	Lon	Alt (m a.s.l)	Installed time	Position
AWS	39.00°	98.88°	4,780	2016	Glacier surface
52,645	38.40°	99.58°	3,320	1959	Regional
52,633	38.80°	98.42°	3,367	1956	Regional
52,657	38.18°	100.25°	2,787	1956	Regional

## Results

### Climate change at the Aug 1st glacier

**Figure 2:**
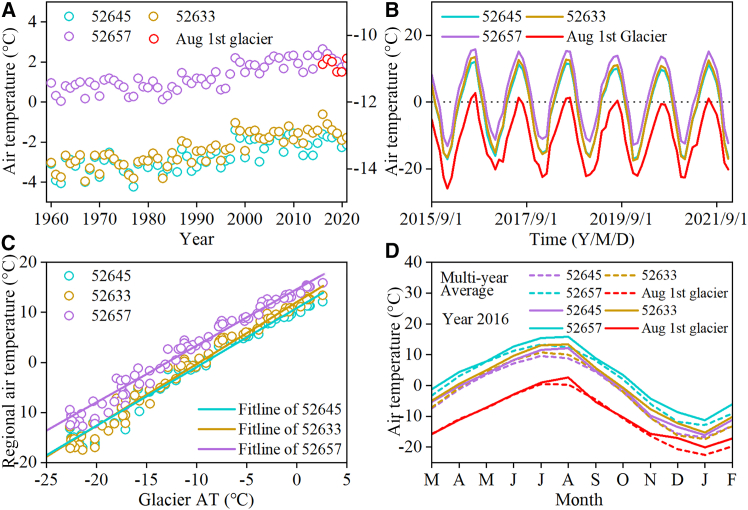
Temperature at Bayi glacier and its surrounding areas (A) Changes in temperature in the region (1960–2021) and on the glacier’s surface (2015–2021). (B and C) Correlation and (C) consistency of glacier air temperature and regional temperature at the monthly scale. (D) Distribution of the monthly temperature in the extreme year (2016) compared with the mean value.

Meteorological observations around the Aug 1st glacier indicate that regional temperatures have increased at a rate of 0.3°C 10^−1^ a^−1^ since 1960 (Figure 2A). Although long-term meteorological observations at the Aug 1st glacier are scarce, a comparison of temperatures at the Aug 1st glacier and surrounding meteorological stations between 2015 and 2021 showed a consistent warming trend in recent years (Figures 2B and 2C). Given the altitude dependence of temperature, the warming rate of the Aug 1st glacier over the past 60 years may be even higher. In 2016, the Qilian mountains experienced the hottest year since 1960, with summer (June–August) and winter (December–February) temperatures 2.2°C and 2.4°C higher, respectively, than the 1960–2021 average in non-glacial areas. In glacier, the surface air temperature in the summer and winter of 2016 were 0.9°C and 2.8°C higher, respectively, than the average. In particular, the temperatures in August and December were 2.3°C and 3.6°C higher, respectively, than the multiyear averages for the corresponding months (Figure 2D).

### Changes in the glacier ablation, glacier surface temperature, and englacial temperature from 2015 to 2018

#### Glacier ablation

The temporal differences in the effects of heatwaves on changes in glacier thickness were pronounced. The measurements showed that in 2016, the period of glacier exposure without sustained snow cover lasted for 52 days, from 28 July to 5 September. During this period, the high temperature days were concentrated from late July to late August, with 33 days when daily air temperature on the glacier exceeded 0°C, accounting for 63% of the total ablation period. Compared to 2016, the ablation season started earlier in 2017, but lasted for a shorter period. Except for ten consecutive days of high temperatures at the beginning of the ablation season, the temperatures fluctuated around 0°C for the rest of the season. In 2017, the glacier was fully exposed for only 44 days, from 13 July to 25 October.

**Figure 3:**
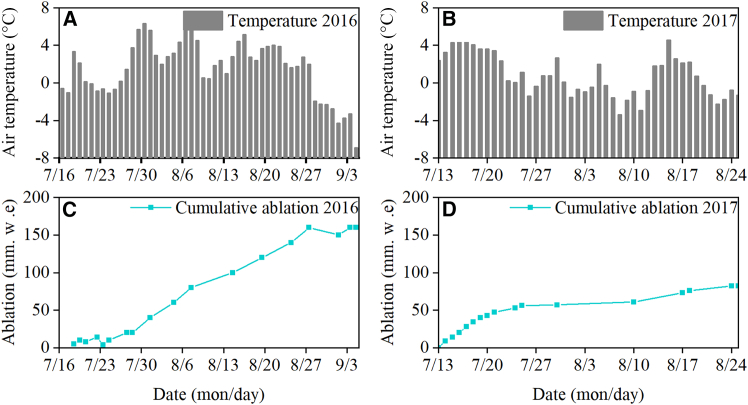
Changes in air temperature and glacier ablation for the Aug 1st glacier during 2016–2017

During the 2016 ablation season, the average daily air temperature of the Aug 1st glacier was approximately 1.53°C (Figure 3A). The glacier thickness decreased by 1.82 m (Figure 3C). During the 2017 ablation season, the mean daily temperature was 0.7°C lower than that during in the same period in 2016, at approximately 0.83°C (Figure 3B). The glacier ablation during this period was approximately 0.82 m (Figure 3D), with a daily ablation rate of 18 mm/day, and more than 80% of the ablation occurring during the 10 days when the temperature was above 0°C.

#### Glacier surface temperature and englacial temperature

There is a lack of long-term monitoring of the glacier temperatures of small glaciers distributed on and around the Qinghai-Tibet plateau. Therefore, although the main focus of this study is to investigate the glacier response to high temperatures through a comparative analysis from 2016 to 2017, this section still presents observational results from all available time periods as much as possible.

#### Daily variations

**Figure 4:**
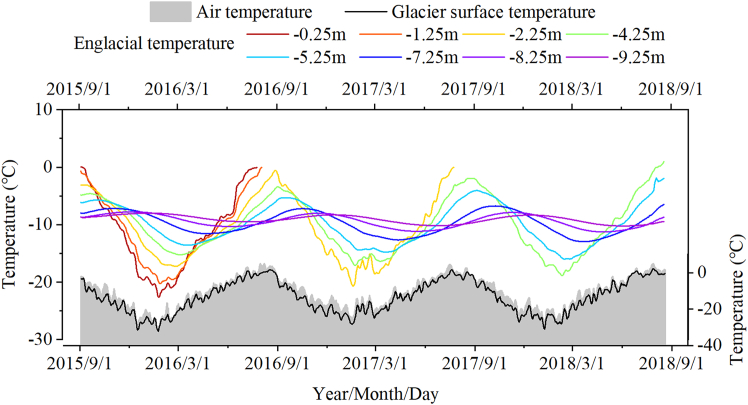
Response processes of the glacier surface temperature and englacial temperature at different depths to air temperature fluctuations

Figure 4 shows the evolution of glacier temperature from 1 September 2015 to 24 August 2018. In line with the observed changes in glacier thickness, the probes initially deployed at depths of −0.25 m and −1.25 m were fully exposed above the ice surface by July 2016, and the probes at −2.25 m were exposed by August 2017. Observations of englacial temperature indicate that fluctuations in glacier surface temperature were almost synchronous with air temperature. However, the glacier surface temperature was generally lower than the air temperature, and the daily air temperature and glacier surface temperature from September 2015 to August 2018 were −10.8°C and −13.4°C, respectively.

In the first year of full observation, the annual englacial temperature was lowest at a depth of −2.25 m, at approximately −9.86°C. From −0.25 m to −2.25 m, the annual difference in englacial temperature was less than 0.1°C. From −2.25 m to −9.25 m, the annual englacial temperature gradually increased with depth, with the annual englacial temperature at −9.25 m being −8.7°C, approximately 1.1°C higher than that at −2.25 m. Within the observed range, the annual englacial temperature was higher than the annual air temperature at all depths, in contrast to the pattern found in other glaciers where the englacial temperature at 10 m was approximately equal to the multi-year average air temperature.

#### Seasonal changes

**Figure 5:**
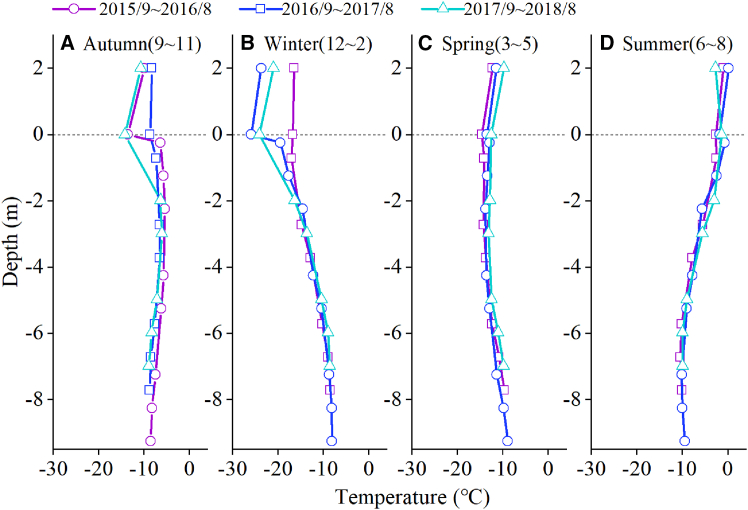
Seasonal variations of air temperature, glacier surface temperature, and englacial temperature from September 2015 to August 2018 Positive values represent elevation above the ice surface, while negative values indicate depth below it.

Figure 5 shows the seasonal variation in englacial temperature. Although the air temperature in the summer of 2016 (Figure 5D, purple) was significantly higher than that in any other year on record, englacial temperature exhibited no significant deviation at any depth during the summer months, and the glacier surface temperature had a weak negative correlation with the air temperature (Figure 5D). A comparison of englacial temperature in autumn 2015 (Figure 5A purple) and 2016 (Figure 5A blue) shows that high summer temperatures did not have a significant effect on ice temperature. The distribution of englacial temperature in winter (Figure 5B) shows that surface ice temperatures were significantly positively correlated with air temperatures. However, the effect of the winter heat wave on englacial temperature did not extend beyond a depth of −2.25 m, and by spring (Figure 5C), the effects of the brief periods of unusually high temperatures gradually dissipated.

## Discussion

### Response of glacier ablation to air temperature

Given their sensitivity to atmospheric forcing, glaciers are considered effective indicators of climate change.^17^ The relationship between glacier ablation and temperature forms the basis of the widely used temperature index models of large-scale glacier ablation.^18^ These models typically assume a constant relationship between glacier ablation and air temperature (or positive cumulative temperature), implying that the parameter of glacier sensitivity to temperature, the degree-day factor (DDF), remains constant.^19^^,^^20^ However, observational studies have suggested that the sensitivity of glacier ablation to climate change may be increasing over time due to changes in glacier surface characteristics. These changes include thinning of the granular snow layer, ice fragmentation,^21^ black carbon accumulation, and increasing ice temperatures, all of which contribute to accelerated glacier ablation.^22^

**Figure 6:**
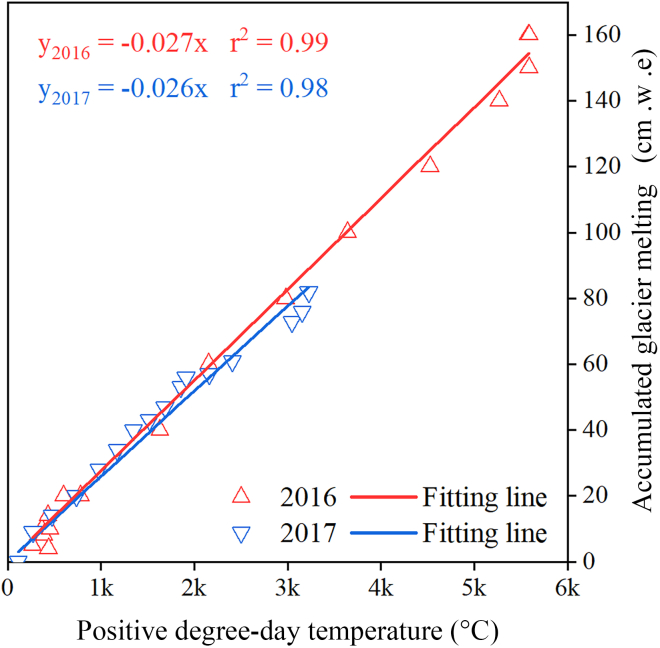
Relationship between glacier mass loss and positive degree-day temperature

In this study, we found that the cumulative glacier ablation of the Aug 1st glacier was significantly and linearly correlated with the cumulative positive temperature (Figure 6). Despite the heat wave in the summer of 2016, which caused the total ablation of the Aug 1st glacier to be twice that of 2017, the slope of the fitted line between the cumulative glacier ablation and cumulative daily positive temperature was almost unchanged. The DDFs for Aug 1st glacier were about 0.026 mm/°C in 2016 and 0.027 mm/°C in 2017. These results suggest that although short-term heat waves can lead to large increases in ablation, the sensitivity of glacier ablation to climate change remains consistent. This finding further demonstrates the applicability of the temperature index model over short time scales.

### Responses of glacier surface temperature to air temperature

Long-term monitoring results from the Alps and polar regions show that continued climate warming has increased the internal ice temperature of glaciers.^2^^,^^3^ As the overall temperature of a glacier increases, the amount of heat required for the glacier to reach the melting threshold decreases.^4^^,^^23^ In addition, previous studies have documented that such warming has transformed cold or polythermal glaciers into temperate glaciers, altering their thermal and mechanical structures and, in some cases, triggering catastrophic ice avalanches.^8^^,^^24^^,^^25^^,^^26^ However, other studies on small glaciers have shown that once the firn layer on the glacier surface completely disappears, glacier melting consumes the most of the heat energy at the glacier surface.^27^ In addition, since the exposed ice surface cannot store meltwater, the heat that would normally be conducted into the glacier via refreezing is lost with runoff, which could lead to a decrease in ice temperature.^7^ Considering the different energy flux processes at the glacier surface and inside the glacier, the responses of glacier surface temperature and englacial temperature to air temperature were analyzed separately in this study.

**Figure 7:**
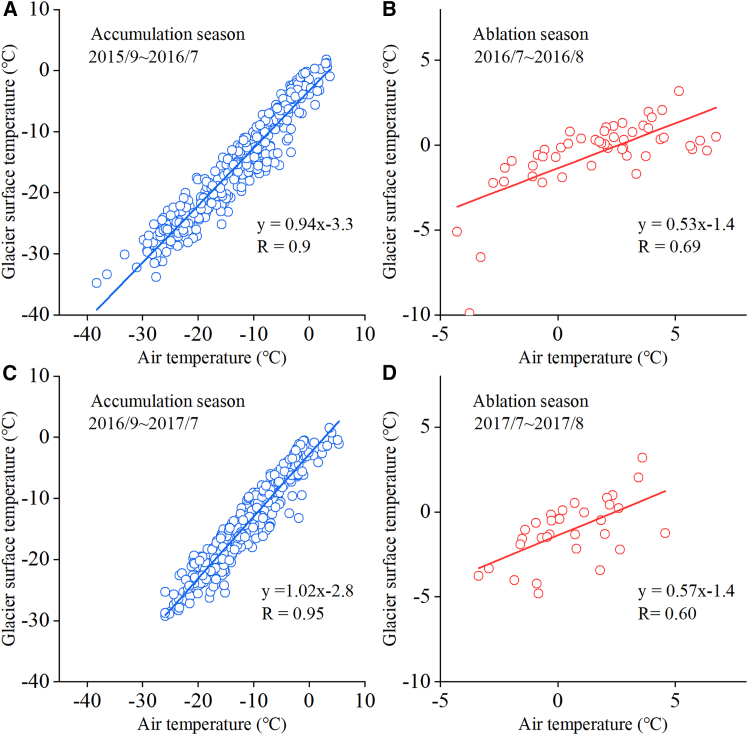
Relationship between glacier surface temperature and air temperature during the ablation and accumulation seasons

The Aug 1st glacier is a small glacier which has completely lost its perennial firn layer. To investigate the response of the ice temperature to climate change, we analyzed the relationships between the glacier surface temperature and air temperature during the ablation and accumulation seasons in different years (Figure 7). The results show that the correlation and slope between the glacier surface temperature and air temperature were much lower during the ablation season than during the accumulation season. From July to August 2016 (Figure 7B), when temperatures were higher, glacier ablation was more intense, but the slope of the surface ice temperature change in response to air temperature was lower. However, from September 2016 to July 2017 (Figure 7C), under the temperature forcing of a warm winter, the change rate of the glacier surface temperature was significantly higher than that of the air temperature. These results indicate that the glacier surface temperature still increased in a positive linear correlation with air temperature, but the rate of increase in the glacier surface temperature was only about 50% of the rate of warming of air temperature.

In theory, glacier surface temperature is influenced by both air temperature and radiation.^28^ However, the response of glacier surface temperature to air temperature during the accumulation season suggests that the thermal state of the glacier surface is primarily controlled by air temperature, with sensible heat transfer dominating the energy process. Compared to the accumulation season, the relationship between glacier surface temperature and air temperature during the ablation season indicates that latent heat consumption during the glacier melting process was the main reason for the suppression of surface warming, as has been confirmed by energy balance mechanism studies.^7^ A study using remote sensing data from 2000 to 2021 shows a significant increasing trend in glacier surface temperature over the Third Pole.^1^ Furthermore, the average glacier surface temperature warming rate across the entire Third Pole has been 0.17°C/10 yr, which is only about half of the regional atmospheric temperature increase rate of 0.38°C/10 yr, consistent with the observational results of this study.^1^

### Responses of englacial temperature to air temperature

#### Lag time

**Figure 8:**
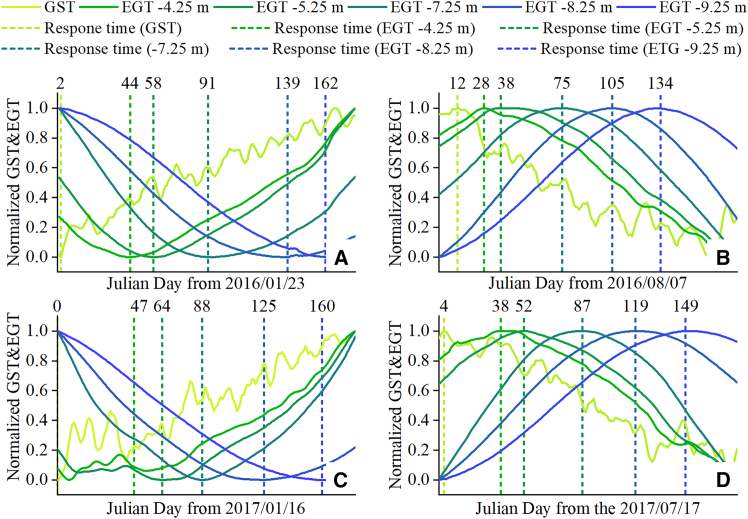
Response time of englacial temperature to extreme air temperature fluctuations (A–E) represent the response of englacial temperature to the coldest air temperature of the year, whereas (B, D) represent its response to the warmest daily air temperature of the year. The dashed lines indicate the time (numbers above the dash line) it took for englacial temperature at different depths to reach the lowest or highest temperatures. In the figure, GST and EGT denote the abbreviations for glacier surface temperature and englacial temperature, respectively.

By comparing the timing of the extremes of englacial temperature and air temperature during 2016–2017, we further assessed the lag in the response of englacial temperature to variations in air temperature at different depths. Specifically, the minimum air temperature was recorded on 23 January, 2016 and 16 January, 2017, while the maximum air temperature was recorded on 7 August, 2016 and 17 July, 2017. The timing of the englacial temperature extremes is shown in Figure 8. In the figure, the englacial temperature depths correspond to the initial depths of the temperature probes (corresponding to the observation depths on 1 September,s 2015).

As shown in Figure 8, the lag time of englacial temperature in response to air temperature is positively correlated with depth. This suggests that the englacial temperature of the Aug 1st glacier is not influenced by abnormal internal warming caused by ice caves or crevasses. Furthermore, significant differences in lag times between warm and cold seasons were observed across different years. In 2016, within the depth range of −4.25 to −9.25 m, englacial temperature responded significantly faster to the “warm” air temperature on 7 August, 2016 than to the “cold” air temperature on 23 January, 2016 (Figures 8A and 8B). Specifically, at depths of −4.25 to −7.25 m, the lag time for englacial temperature to respond to the highest air temperature was 15–20 days shorter than that for the lowest air temperature. At greater depths of −8.25 to −9.25 m, the lag time for high temperatures was about 30 days shorter than for low temperatures.

In 2017, the englacial temperature observation depths were 1.62 m shallower than in 2016 due to surface ablation in 2016. Assuming no significant differences in external air temperature, the lag time of englacial temperature to air temperature in 2017 would be expected to be shorter than in 2016. However, as shown in Figures 8C and 8D, the results reveal a different pattern. While the englacial temperature at the surface and at initial depths of −8.25 m and −9.25 m responded fast to the air temperature on 16 January, 2017, the lag time of englacial temperature at other depths was generally longer in 2017 than in 2016. In addition, the seasonal difference in the englacial temperature lag time in 2017 was smaller compared to 2016. For example, within the depth range of −4.25 to −8.25 m, the lag time difference between the highest and lowest air temperature was only about 3–6 days. Even at a depth of −9.25 m, the lag time difference between the warm and cold seasons was only about 10 days.

The englacial temperature lag times at different times in the Aug. 1st glacier demonstrated that surface englacial temperature (approximately equal to glacier surface temperature) exhibits a markedly longer response time to high temperatures than to low temperatures. Furthermore, the englacial temperature lag time increases with increasing air temperature. However, the lag time of surface englacial temperature to low temperatures does not show significant interannual differences. Observations from 2016 to 2017 indicate that glacier melt likely contributes to these patterns. When the glacier surface reaches 0°C, melting consumes latent heat, which reduces the net heat diffusion into the ice. Small mountain glaciers without a firn layer lack conditions for meltwater infiltration and refreezing at the surface, potentially delaying heat transfer to the glacier interior during prolonged summer warmth.

**Figure 9:**
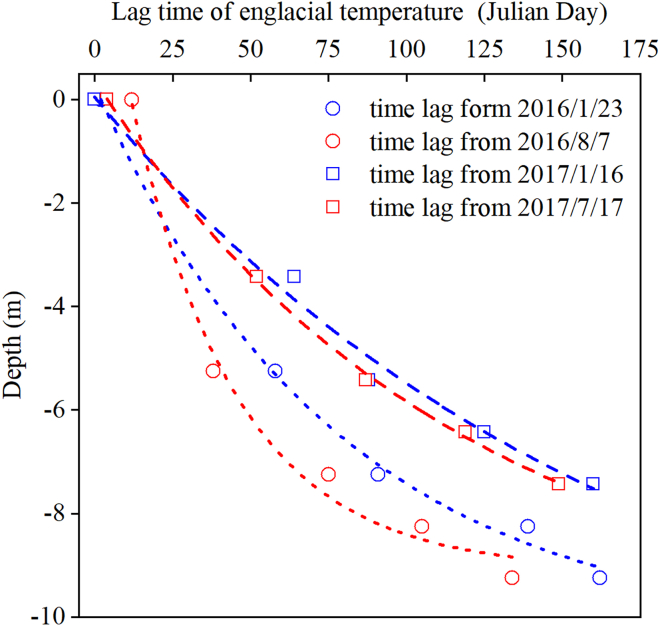
Lag time of englacial temperature in response to air temperature at different depths

In addition to the earlier mentioned effects of melting on the total heat that eventually enters the interior of the glacier, the internal englacial temperature to air temperature is also influenced by two additional factors. First, based on Fourier’s law, the temperature gradient between the interior and exterior of the glacier, as well as between different layers within the glacier, is directly proportional to the rate of heat conduction.^29^ Second, the initial temperature of the ice inversely affects the rate of heat conduction. This is because the thermal diffusivity, which determines the rate of heat transfer, decreases as the ice temperature increases, leading to slower heat conduction at higher ice temperatures.^30^ After correcting for the actual observed depths of englacial temperature, we reconstructed the lag time distributions of englacial temperature at different depths for 2016 and 2017 (Figure 9).

The results indicate that lower summer temperatures and higher winter temperatures correspond to longer englacial temperature lag times. Furthermore, the exponential relationship between englacial temperature lag time and depth indicates that the change rate of lag time decreases with depth. All of the earlier mentioned phenomena indicate that current glacier internal heat conduction is primarily influenced by temperature gradients rather than by the ice thermal diffusivity. This is because lower summer temperatures, higher winter temperatures, and the ice temperature distribution at greater depths all represent smaller temperature gradients.

#### Vertical distribution

**Figure 10:**
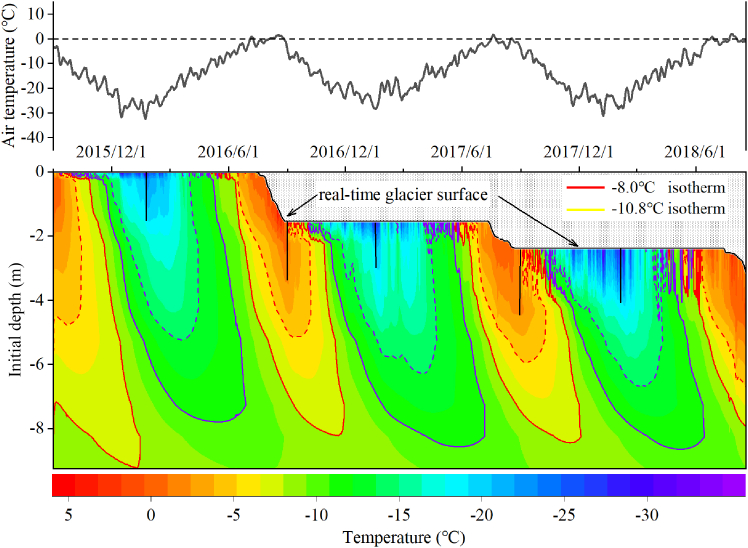
Distribution of air temperature, englacial temperature and glacier surface changes at the Aug 1st glacier from 2015 to 2018 Depths in the figure refer to initial ice temperature monitoring depth in September 2015. The −8.0°C and −10.8°C isotherms are highlighted to clearly illustrate the coordinated variations between ice temperature and glacier ablation.

Differences in the lag time of the englacial temperature response to temperature at different depths result in variations in its vertical distribution. Based on the relationship between glacier melt and positive degree-days in Figure 6, we reconstructed the glacier surface change. On this basis, we excluded invalid data (the temperature probes originally embedded in the ice were completely exposed at the glacier surface due to ablation) and interpolated the englacial temperature distribution of the Aug 1st glacier. Figure 10 shows the englacial temperature distribution over a 36-month period, including two complete ablation-accumulation cycles from 2016 to 2017. During the observation period, the air temperature at the glacier surface varied between −37.8°C and 13.0°C, with an average temperature of −10.6°C, and the surface mass loss of the glacier exceeded 3 m (Figure 10).

Theoretically, if the response of englacial temperature to air temperature is continuous and timely, the depth of propagation of both “cold energy” in winter and “heat energy” in summer into the glacier relative to the contemporaneous glacier surface would be consistent under the same climatic conditions. This implies that for any given isotherm, its maximum depth would progressively extend into the interior of the glacier each year, with the extent of this deepening equal to the ablation thickness of the glacier surface from the previous year. As shown in Figure 10, the interannual variation in depth of shallow englacial temperature isotherms is not significant. In particular, for isotherms >−5°C (dark red) and <−20°C (dark blue), although the profiles of these isotherms show noticeable interannual differences, their maximum depths relative to the real-time glacier surface remain almost constant, fluctuating around 3 m below the glacier surface. However, the depth of the englacial temperature distribution below the active layer shows a limited response to air temperature and surface melt. Taking the −10.8°C isotherm as an example, the maximum depth of the −8.0°C isotherm was significantly shallower than in 2015, despite higher summer air temperatures and thinner glacier thickness in 2016 compared to 2015. Furthermore, even with 2.4 m of surface ice ablation during 2016–2017, the maximum depth of the −8.0°C isotherm did not change significantly during this period. Similarly, during the winters of 2015–2017, the maximum depth of the −10.8°C isotherm showed a downward deepening trend. However, the extent of this deepening was significantly less than the ablation of the glacier surface.

The influence of ambient temperature on englacial temperature gradually diminishes with increasing depth. In the upper part of the active layer, englacial temperature responds more promptly to air temperature due to the shorter heat transfer distance. Relative to the real-time glacier surface, the interannual differences in englacial temperature distribution in the upper active layer (<3 m) are not significant. However, in the lower active layer, the shortened heat transfer distance after surface ablation does not result in a rapid change in the englacial temperature distribution. The variations in englacial temperature at different depths ultimately lead to a continuous thinning of the glacier’s active layer. This reduces the heat storage capacity of the glacier in its upper layers and weakens its cooling protection, further accelerating surface ablation. As a result, the glacier struggles to reach a new equilibrium, which has a direct impact on its long-term stability.

### Interaction between the glacier temperature and glacier ablation

#### The impact of firn layers on glacial temperature and ablation

The acceleration of glacier ablation is primarily driven by climate change, with rising air temperatures being the direct cause. Understanding how glaciers respond to factors such as temperature, precipitation, and radiation has been a central focus of research, and these mechanisms have become relatively well understood.^20^ More recently, as the effects of rising temperatures on glacier ablation have been studied in more detail, the self-feedback mechanisms within glacier systems driven by changes in glacier properties have attracted increasing attention.^22^ Among these, the increase in englacial temperature and its role in enhancing glacier ablation is particularly critical.^20^

To accurately account for the mutual feedback between glacier ablation and englacial temperature increase, researchers have studied different glacier types in regions such as the polar regions,^3^^,^^5^ the Alps,^2^^,^^27^ and the Tibetan plateau.^7^ These studies indicate that snow and ice structure at the glacier surface is a key factor influencing ablation and changes in englacial temperature distribution. Observations show that for glaciers with firn layers, englacial temperature exhibits an increasing trend.^10^^,^^20^ Notably, the direct contributions and range of influence of atmospheric sensible heat and solar radiation on englacial temperature increase are limited.^26^ Instead, meltwater infiltration and refreezing have been identified as the primary drivers of englacial temperature increase in such glaciers.^9^

Once the infiltration depth of meltwater exceeds the infiltration depth of winter cold air, glaciers undergo abrupt and irreversible changes in their properties.^8^^,^^26^ Changes in englacial temperature reflect the cumulative effects of long-term climate change. Although these changes lag behind air temperature increases,^22^ the englacial temperature increases driven by meltwater may exceed the rate of climate warming.^2^

Under similar climatic conditions, higher englacial temperature reduces the energy required for ice to reach the critical melting temperature of 0°C,^2^^,^^4^ resulting in longer daily melt durations, extended ablation seasons, and greater ablation volumes in a given time period.^4^ As heat travels from the surface to the base of the glacier,^3^^,^^8^ glaciers warm overall, leading to the transformation of cold and polythermal glaciers into temperate glaciers.^24^^,^^25^ This transformation affects glacier rheology and basal conditions, further accelerating ablation.^9^^,^^31^ Examination of the relationship between glacier ablation and englacial temperature suggests that, driven by climate warming, accelerated ablation and rising englacial temperature have formed a mutually reinforcing positive feedback loop. This cycle will further accelerate glacier ablation rates in the future.

#### How do englacial temperature and ablation interact after the disappearance of the firn layer?

It is widely accepted that the continued accumulation of energy within glaciers lowers the temperature threshold required for glacier melt, thereby facilitating further melting. However, this phenomenon only occurs where firn layers remain on polar ice caps or large mountain glaciers. Studies of englacial temperatures in ablation zones by Wang et al.^7^ and Signer^27^ suggest that in the absence of snow cover on the glacier surface, reduced meltwater infiltration can cause a temporary decrease in ice temperature. However, once the physical properties of bare ice change under long-term warming stress to meltwater infiltration, a positive feedback loop between glacier ablation and increasing ice temperature will be re-established.^7^

**Figure 11:**
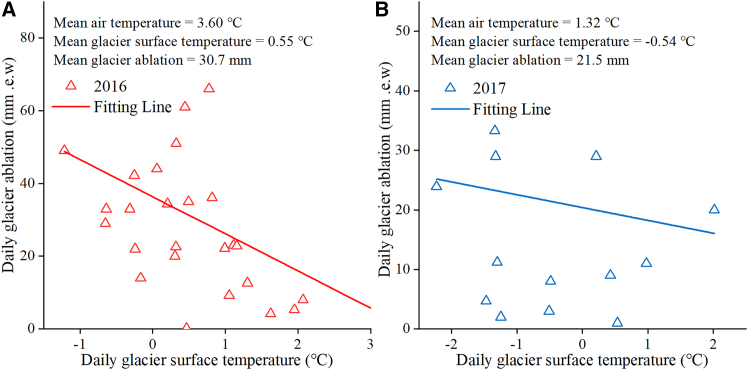
Relationship between daily glacier surface temperature and daily glacier ablation

This study has systematically investigated the interannual and seasonal variations of glacier ablation, glacier surface temperature and englacial temperature, and their respective responses to air temperature. The results provide preliminary evidence for non-linear responses of glacier melt and englacial temperatures to air temperature. Here, we further analyze the correlation between daily glacier ablation and glacier surface temperature. The results indicate that the glacier surface temperature was negatively correlated with daily ablation during the ablation period of the Aug 1st glacier (Figure 11). The linear slope of ablation versus glacier surface temperature was larger in 2016, when temperatures were higher and ablation was more intense (Figure 11A). However, in both years, glacier ablation suppressed the increase in ice temperature in response to air temperature, but ice temperature did not decrease on the Aug 1st glacier (Figure 7). The slope of the linear correlation between cumulative glacier ablation and cumulative temperature did not change significantly (Figure 6), despite the fact that both air temperature and glacier surface temperature were much greater during the ablation period in 2016 than in 2017. This finding also suggests that short-term changes in ice temperature do not have a direct and significant effect on glacier ablation.

Due to the limited observation period of englacial temperature within the active layer in this study, we cannot directly assess the feedback relationship between englacial temperature and glacier ablation in Aug 1st glacier. However, based on the changes in the lag time and vertical distribution of englacial temperature, we summarize that glacier ablation affects englacial temperature in the following ways: (1) latent heat from ice melting consumes some of the energy reaching the glacier surface; (2) glacier ablation leads to the loss of heat that has already entered the glacier and is stored in the surface ice; and (3) due to the lack of infiltration and refreezing conditions (mainly in the firn layer), meltwater drains rapidly from the glacier surface. Consequently, the energy losses from processes (1) and (2) do not return to the glacier. These processes not only reduce the total heat transfer from the surface to the interior of the Aug 1st glacier, but also disrupt the continuous pathway for heat input, ultimately mitigating the impact of air temperature on deeper englacial temperature. In addition, rapid ablation coupled with delayed changes in ice temperature can lead to thinning of the glacier’s active layer, weakening its ability to buffer external heat.

Although glacier ablation temporarily suppresses englacial temperature increases, the cumulative effects of long-term climate change are expected to increase the feedback from glaciers to global warming over time. Nonetheless, understanding the mechanisms and patterns of the ice temperature ablation, as well as glacier stability and climate sensitivity, remains limited and require a multidisciplinary approach including observations, modeling, and analysis.

### Conclusion

Between 2016 and 2017, the Aug 1st glacier exhibited significant interannual variations in ablation and ice temperature. Summer ablation reached 1.60 m in 2016 and decreased to approximately 0.8 m in 2017. The DDFs were 0.026 mm/°C and 0.027 mm/°C, indicating stable sensitivity to short-term climate variations. Glacier surface temperature was positively correlated with air temperature; however, the warming rate during the ablation season was only about half that of the accumulation season, and englacial temperature showed depth-dependent and seasonally varying lags behind air temperature. Latent heat consumption and rapid meltwater runoff partially mitigated surface warming, while thinning of the active layer reduced heat input to deeper ice. Despite these processes, the overall short-term response of glacier ablation to air temperature remained consistent.

### Limitations of the study

The limitations of this study, including the short observation period and the lack of precipitation data, constrain the ability to fully characterize and model the feedbacks between ice temperature and glacier ablation. Long-term and comprehensive observations are therefore required to improve understanding of these processes and to enhance the accuracy of glacier response assessments under changing climatic conditions.

## Resource availability

### Lead contact

Further information and requests for resources and reagents should be directed to and will be fulfilled by the lead contact, Rensheng Chen (crs2008@lzb.ac.cn).

### Materials availability

This study did not generate new unique reagents.

### Data and code availability

• The datasets generated and analyzed during this study are not publicly available due to institutional data confidentiality policies but can be obtained from the corresponding author upon reasonable request.
• No code was generated or used in this study.
• Any additional information required to reanalyze the data reported in this paper is available from the lead contact upon request.

## Acknowledgments

We are very thankful to the anonymous reviewers for their comments and suggestions helped improve the manuscript. This work was supported by the 10.13039/100014718National Natural Sciences Foundation of China (42401143), the 10.13039/100014718National Natural Sciences Foundation of China (42271154), the 10.13039/100014472Scientific Research Fund of Hunan Provincial Education Department (22A0497), and the Provincial 10.13039/501100004735Natural Science Foundation of Hunan (2025JJ60225).

## Author contributions

All authors contributed to the study conception and design. Material preparation, data collection and analysis were performed by G.L., R.C., C.H., J.L., Z.L., Y.Y., S.G., X.W., Y.L., and C.G. The first draft of the manuscript was written by G.L. and all authors commented on previous versions of the manuscript. All authors read and approved the final manuscript.

## Declaration of interests

The authors declare that they have no known competing financial interests or personal relationships that could have appeared to influence the work reported in this paper.

## STAR★Methods

### Key resources table

**Table:** 

REAGENT or RESOURCE	SOURCE	IDENTIFIER
**Deposited data**		

Raw and analyzed data	This paper	https://hhsy.casnw.net/

**Software and algorithms**		

ArcGIS10.2	Esri	https://desktop.arcgis.com/zh-cn/desktop/
MATLAB R2016a	Mathworks	https://www.mathworks.com/products/matlab.html

### Experimental model and study participant details

This study does not involve any living participants.

### Method details

#### Air temperature observation

To study the response of the cryosphere to climate change, a comprehensive automatic weather station (AWS) was installed by the Northwest Institute of Eco-Environment and Resources, Chinese Academy of Sciences (NIEER-CAS, https://hhsy.casnw.net/index) in 2016 (Figure 1B). In this study, we focused on the energy fluxes at the glacier surface and within the glacier, which leads to glacier melting and warming. At the AWS, the air temperature at 2 m above the ice surface was monitored via a Vaisala HMP 155A temperature sensor with a temperature measurement accuracy of ±0.17°C.

Owing to the short duration of the AWS observations, we used data from three national weather stations (from the CMA, https://data.cma.cn/data) as a reference to study the long-term climate change of the Aug 1st Glacier. Details of all 4 weather stations are given in Table 1.

#### Glacier ablation observation

Automated photographic equipment was used to monitor the glacier ablation. The equipment included a series of aluminum stakes painted with different colors or marked with rubber tape to record height changes of the glacier surface. In addition, a camera was placed approximately 1 m away to record surface changes of the glacier. The camera was set to take pictures automatically every two hours from 7:00 to 19:00 daily. Due to the fragmented surface of the glacier during the ablation season and the reliance on visual interpretation for data collection, some measurement uncertainty exists in the glacier ablation. However, through field comparisons, the error range is approximately 0.5 cm/day.

#### Glacier surface temperature and englacial temperature observation

Glacier surface temperature was measured using SI-111 sensors with an accuracy of ±0.2°C. To monitor englacial temperature changes, a temperature chain was installed around the AWS. The englacial temperature borehole was drilled by steam injection with a hole diameter of approximately 5 cm and a depth of 9.5 m. The sensor was initially installed at depths of -0.25 m, -1.25 m, -2.25 m, -4.25 m, -5.25 m, -7.25 m, -8.25 m, and -9.25 m. All depths are referenced downward from the ice surface, denoted as negative values. The surface temperature was measured by an Apogee infrared radiometer with a measurement accuracy of ±0.2°C∼±0.5°C. All glacier surface temperature and englacial temperature data were collected by a solar-powered CR1000 data logger, with a monitoring interval of 30 minutes. The temperature chain was first installed in the summer of 2015 and data were collected from 1 September 2015 after the borehole was completely refrozen.

### Quantification and statistical analysis

#### Division of calendar year and seasons

To account for the freeze–thaw cycle of glaciers, the calendar year in this study was defined as beginning in March of the current year and ending in February of the following year. Accordingly, the seasons were defined as spring (March–May), summer (June–August), autumn (September–November), and winter (December–February of the following year).

#### Observation depth of englacial temperature

The initial depths of the ice temperature probes changed as the glacier surface ablated. Because ice temperature was distributed non-linearly across different layers and heat conduction exhibited a significant time lag between them, we did not interpolate or fill missing data before conducting further analyses. All depths are referenced downward from the ice surface, denoted as negative values. All depths referenced are relative to the surface elevation on 1 September 2015, unless otherwise noted. When a temperature probe was fully exposed on the glacier, the temperature data were recorded as *Nodata*.

#### Data statistics and analysis

Air temperature, ablation, glacier surface temperature, and englacial temperature trends were directly obtained from observational data. The englacial temperature distribution in Figure 10 was derived using linear interpolation. Correlation analyses among air temperature, ablation, glacier surface temperature, and englacial temperature were performed through linear correlation methods.
